# Biological Potential of Polyethylene Glycol (PEG)-Functionalized Graphene Quantum Dots in In Vitro Neural Stem/Progenitor Cells

**DOI:** 10.3390/nano11061446

**Published:** 2021-05-29

**Authors:** Yunseong Ji, Yu-Meng Li, Jin Gwan Seo, Tae-Su Jang, Jonathan Campbell Knowles, Sung Ho Song, Jung-Hwan Lee

**Affiliations:** 1 Institute of Tissue Regeneration Engineering (ITREN), Dankook University, 119 Dandae-ro, Cheonan 31116, Korea; jys1432@naver.com (Y.J.); wendyleeym0925@gmail.com (Y.-M.L.); 2 Department of Nanobiomedical Science & BK21 PLUS NBM Global Research Center for Regenerative Medicine, Dankook University, 119 Dandae-ro, Cheonan 31116, Korea; 3 Division of Advanced Materials Engineering, Kongju National University, Cheonan 32588, Korea; wlsrhks0324@smail.kongju.ac.kr; 4 Department of Pre-medi, College of Medicine, Dankook University, Cheonan 31116, Korea; jangts@dankook.ac.kr; 5 UCL Eastman-Korea Dental Medicine Innovation Centre, Dankook University, 119 Dandae-ro, Cheonan 31116, Korea; j.knowles@ucl.ac.uk; 6 Division of Biomaterials and Tissue Engineering, Eastman Dental Institute, University College London, London WC1E 6HH, UK; 7 The Discoveries Centre for Regenerative and Precision Medicine, Eastman Dental Institute, University College London, London WC1E 6HH, UK; 8 Department of Regenerative Dental Medicine, College of Dentistry, Dankook University, Cheonan 31116, Korea

**Keywords:** cytotoxicity, polyethylene glycol functionalized-graphene quantum dots (PEG-GQDs), neural stem/progenitor cells (NSPCs), biocompatibility, the visible bio labeling system

## Abstract

Stem cell therapy is one of the novel and prospective fields. The ability of stem cells to differentiate into different lineages makes them attractive candidates for several therapies. It is essential to understand the cell fate, distribution, and function of transplanted cells in the local microenvironment before their applications. Therefore, it is necessary to develop an accurate and reliable labeling method of stem cells for imaging techniques to track their translocation after transplantation. The graphitic quantum dots (GQDs) are selected among various stem cell labeling and tracking strategies which have high photoluminescence ability, photostability, relatively low cytotoxicity, tunable surface functional groups, and delivering capacity. Since GQDs interact easily with the cell and interfere with cell behavior through surface functional groups, an appropriate surface modification needs to be considered to get close to the ideal labeling nanoprobes. In this study, polyethylene glycol (PEG) is used to improve biocompatibility while simultaneously maintaining the photoluminescent potentials of GQDs. The biochemically inert PEG successfully covered the surface of GQDs. The PEG-GQDs composites show adequate bioimaging capabilities when internalized into neural stem/progenitor cells (NSPCs). Furthermore, the bio-inertness of the PEG-GQDs is confirmed. Herein, we introduce the PEG-GQDs as a valuable tool for stem cell labeling and tracking for biomedical therapies in the field of neural regeneration.

## 1. Introduction

Stem cell based therapy is currently a promising and advanced research field that attracts attention as a critical player for the treatments of intractable diseases, such as cancer, neurological disorders, cardiac diseases, diabetes, and liver diseases [1,2]. There is a strong need for stem cell based treatment methods due to the limited self-repair and regeneration ability of nerve tissue, especially in neurological disorders including spinal cord injury and traumatic and ischemic brain injury. These disorders have very high morbidity, which can easily cause disability with lifelong economic and emotional cost or even death [3,4]. Generally, stem cells or stem cell derived neural cells are implanted to the injury site. These transplanted cells directly replaced the host’s dead neural cells and secreted neurotrophic factors to promote neural regeneration. Neural stem/progenitor cells (NSPCs), a subtype of multipotent stem cells, can self-renew and generate neurons and glia in the nervous system. Exogenous transplantation and endogenous regeneration of NSPCs in damaged nerve tissue are promising therapies in treating the aforementioned neurological disorders [5,6]. However, in many instances, the scientific rationale and preclinical efficacy remains obscured due to the problematic tracking and monitoring of transplanted stem cells. Research of these biological processes necessitates affordable analytical tools with high resolution, high sensitivity, and the capability to perform multiplexed analysis. In past research fluorescent probes, such as organic fluorophores, were used to discover the scientific evidence for the therapeutics [7]. However, cytotoxicity and the alteration of biological effects to the cells caused by unknown interaction with materials influence the efficacy of stem cell therapies. Thus, it is desirable to develop biocompatible and biologically inert materials. With these markers, the accuracy of the investigation of the stem cell’s behavior after transplantation in the nerve tissues will be improved and successful evaluation of the progress will exclude interference caused by the materials.

Quantum dot (QD) is a promising solution for more capable bioassay and bioimaging technology. QDs emerged as a result of the discovery that the size of the nanomaterials determines the properties of QDs [8,9]. In terms of optical properties, QDs are superior in almost all respects compared to organic dyes [7]. Their size-dependent tunable emission wavelength and narrow spectrum renders them more distinct from the biological autofluorescence signals. However, the application of QDs in living cells has been restricted by the dissolution of heavy metal ions such as cadmium. One of the alternative candidates, graphene quantum dots (GQDs), which are a subset of nano-carbon quantum dots without concerns about the dissolution of heavy-metal ions, exhibited excellent photo-luminescent (PL) and photostable properties [10,11,12,13]. Therefore, GQDs are now being used in bioimaging, biomedical diagnostic, and therapeutic applications. Furthermore, many studies have reported that GQDs were well composited with anti-cancer medicines, cancer cell-sensing, and cancer-targeting molecules. These bio-conjugated GQDs showed enhanced cellular uptake and effectively tracked the delivery and release of bio-molecules in cancer cells [14,15,16,17]. Hanjun Sun et al. also reported that GQDs alleviated wound infection and reduced the production of biofilms in implants of a mouse wound model [18]. GQDs based therapies were also highlighted in the neural regeneration field. Songhua Xiao et al., reported that the GQDs-neuroprotective peptide effectively inhibited the aggregation of Aβ1-42 and α-synuclein (α-syn) and rescued the brain neural cells [19]. Moreover, GQDs were also found to protect neurons and oligodendrocytes from T cell mediated damage and saved neuronal death and synaptic loss from the toxicity of aggregated α-syn without conjugating with neuroprotection drugs [20]. However, there are few reports on the bioimaging of GQDs in stem cell therapies towards neural regeneration.

Recently, the cytotoxicity related to the surface functional group and the size which influences the biological effects and the uptake of GQDs were addressed. Weihu Shang et al. showed that GQDs smaller than 10 nm demonstrated direct and easy penetration into stem cells without affecting their viability, proliferation, or differentiation capacity [21]. Furthermore, Yu Chong et al. described the low cytotoxicity of ultra-small-sized high oxygen content GQDs made by the chemical oxidation method [22]. On the other hand, in the study of Yichun Xie et al., the three different functional groups were used to investigate cytotoxicity. They concluded that the -COOH functionalized GQDs are more biocompatible compared to NH_2_ or OH functionalized ones. However, all three different surface chemistry influenced the biological pathway, such as phosphorylation [23]. Therefore, research about particles completely inert inside the body is recommended. One of the candidates is polyethylene glycol (PEG). The PEGs are a FDA approved non-toxic material that is frequently used in many biomedical applications, including bioconjugation [24], drug delivery [25,26], and surface functionalization [27]. PEG functionalized materials showed improved solvation property, increased drug delivery efficiency, and prolonged circulation time in both in vitro and in vivo studies [28,29,30,31]. Moreoever, PEGylated nanoparticles are resistant to protein adhesion and biodegradation [32,33,34].

For these reasons, the PEG-GQDs were fabricated and investigated as a platform of the theranostic agents for the vulnerable and fragile nerve tissues. The GQDs were fabricated by the graphite intercalation compounds mediated exfoliation method and the PEGylation proceeded on the GQDs’ surface through the solvothermal method. The size, surface charge, surface functional groups, and photoluminescence property of GQDs before and after PEGylation were investigated. Then, the experiments that dealt with the concentration dependant cytotoxicity established the available dose of material when the fetal rat NSPCs were employed. Moreoever, it was confirmed that the PEG-GQDs are well internalized into the cell. Finally, the presence of the PEG-GQDs’ influences on cell differentiation was closely investigated. Through this study, a method to utilize the low-cytotoxic, bio-inert, and bioimaging nanoparticle is proposed.

## 2. Materials and Methods

### 2.1. Graphene Quantum Dot Preparation and Characterization

The GQDs were fabricated by the graphite intercalation compounds (GICs) method described previously [35,36,37]. Briefly, a potassium sodium tartrate (KNaC_4_H_4_O_6_·4H_2_O, Sigma Aldrich, Burlington, MA, USA) precursor was introduced in the graphite by mixing and the exfoliation proceeded at 250 °C for 24 h. The size distribution of the GQDs was controlled by sieving with a dialysis membrane (10,000 and 8000 NMWL, Amicon Ultra-15, Sigma Aldrich, Burlington, MA, USA). The particles were dried for several days under vacuum conditions. The surface functional groups of GQD were reacted with polyethylene glycol (PEG-bis(amine), MW: 2000, Sigma Aldrich, Burlington, MA, USA) via an amide bond [38]. The aqueous solution of GQDs (0.5 mg/mL) was mixed with PEG-bis(amine) in a ratio of 2 mg PEG per mL of solution. The anchoring process was performed at 120 °C for 1 h via a hydrothermal method.

X-ray diffractometer (XRD; Rigaku, UltimaIV, Tokyo, Japan, equipped with a Cu Kα source), dynamic light scattering and zeta potential (DLS; Zetasizer Nano ZS, Malvern Instruments, Malvern, WR14 1XZ, UK, solvent: DW), X-ray photoelectron spectroscopy (XPS; Sigma Probe, Al Kα sources, Thermo Fisher Scientific, Waltham, MA, USA), transmission electron microscopy (TEM, Tecnai G2 F 30, FEI, Hillsboro, OR, USA), Fourier transform infrared spectroscopy (FT-IR; Varian 670-IR, Varian, Palo Alto, LA, USA, equipped with ATR accessories from 400 and 4000 cm^−1^) analyses were conducted.

### 2.2. Visualization Ability of PEG-Graphene Quantum Dots (PEG-GQDs)

PEG-GQDs were dissolved in the sterilized deionized water and diluted to create different concentrations (0, 10, 40, 80, 160, 320, 640, and 1280 µg/mL). The amount of 100 µL PEG-GQDs was added to the 96-well plate and fluorescence images were taken by a fluorescence microscope (Olympus IX7151, Tokyo, Japan).

### 2.3. Neural Stem/Progenitor Cells (NSPCs) Culture

Neural stem/progenitor cells (NSPCs) were prepared using a previously reported method [39,40]. The Animal Care and Use Committee of Dankook University approved all the animal care and experimental procedures (Approval No. 18-032). Briefly, the telencephalons were isolated from the Embryonic day 14.5 Sprague-Dawley rats. The tissue was washed twice with 3 mL Hank’s balanced salt solution (HBSS) (WELGENE, Gyeongsan-si, Korea, LB 003-02) and complete growth media, respectively. A single cell suspension was dissociated in the growth media by using only one 1 mL pipette tip. After being set down for 1 min, the suspension was filtered with a 40 µm filter. The amount of 5 × 10^5^ rNSPCs was seeded into a 6 cm low-attachment culture dish and incubated in a 37 °C, 5% CO_2_, and 100% humidity incubator. Every 2 days, half of the media was replaced with a fresh one. Cells were passaged once a week when the neurospheres were larger than 200 µm in diameter. For consistency, cells between passage 2 and 5 were used in this study.

The complete growth media contains DMEM: F12 (Gibco, Grand Island, NY, USA, 11320-033), L-GlutaMAX (2 mM, Gibco, Grand Island, NY, USA, 35050061), 2% B27 minus vitamin A (Gibco, Grand Island, NY, USA, 12587010), 1% Penicillin/ Streptomycin (Gibco, Grand Island, NY, USA, 15140122), 20 ng/mL epidermal growth factor (Peprotech, Rocky Hill, NJ, USA, AF-100-15), and 20 ng/mL basic fibroblast growth factor (Peprotech, Rocky Hill, NJ, USA, 100-18B).

The differentiation media contains Neurobasal plus medium (Gibco, Grand Island, NY, USA, A3582901), 2% B27 Plus Supplement (Gibco, Grand Island, NY, USA, A3582801), 0.5 mM L-GlutaMAX, and 1% Penicillin/Streptomycin.

For identification of the differentiation potential of rNSPCs, cells were firstly seeded in the 96-well plate and incubated for 24 h under the growth media. Then, the growth media was changed into differentiation media to induce the differentiation of rNSPCs and cells were cultured for another 7 days. The media was changed every 2–3 days.

### 2.4. Cell Viability and Cytotoxicity Cssays

For the culture of NSPCs, the plates were coated with 1% Matrigel (Corning, Bedford, MA, USA, 356234), a good basement membrane biomaterial. In detail, the original Matrigel was diluted to 1% with ice-cold DMEM/F12 media. The amount of 100 µL of 1% Matrigel solution was added to the 96-well plate and the plate was then placed into an incubator at 37 °C for 1 h. Before seeding cells, the plate was gently washed once with DMEM/F12 media. The amount of 1 × 10^4^ rNSPCs in 100 µL growth media was seeded in a precoated 96-well plate and permitted to grow for 24 h. Subsequently, different concentrations of PEG-GQDs (0, 10, 40, 80, 160, 320, 640, and 1280 µg/mL) were added to the cells. Twenty-four hours later, after discarding the supernatant, cells were carefully washed once with warm PBS and treated with CCK-8 reagent (Dojindo, Tokyo, Japan, CK04-20) diluted with prewarmed media and incubated for another 2 h. Then the absorbances were measured with a microplate reader at 450 nm. For detecting the viable cells, the LIVE/DEAD™ Viability/Cytotoxicity Kit (Invitrogen, Eugene, OR, USA, L3224) was used. The cells were incubated with Calcein AM (2 µM) and Ethidium homodimer-1 (4 µM) and were diluted with prewarmed DMEM: F12 media for 30 min at 37 °C. Before capturing, cells were washed once with prewarmed DMEM: F12 media. Images were captured with a fluorescence microscope (Olympus IX7151, Tokyo, Japan) at 40×.

### 2.5. Cellular Visualization Ability of PEG-GQDs

To check the visualization ability and intracellular location of PEG-GQDs, confocal laser-scanning microscopy (Zeiss LSM 700, Carl Zeiss, Oberkochen, Germany) was used. The rNSPCs were seeding on the 1% Matrigel pre-coated 15 mm cover-glasses (5 × 10^4^ cells in 500 µL growth media) in a 24-well plate and incubated for 24 h. PEG-GQDs were added to the cells at a concentration of 320 µg/mL. After 24 h of incubation, the supernatant was removed and cells were washed twice with PBS to remove residual PEG-GQDs. Subsequently, the cells were fixed with 4% paraformaldehyde (PFA) (T&I, Chuncheon, Korea, BPP-9004) for 30 min, following three times of PBS washing. For better observation of cell morphology, Alexa Flour 488 Phalloidin (Invitrogen, Eugene, OR, USA, A12379) solution was added to cells and incubated for another 30 min at room temperature. After washing it three times in PBS, the fluorescence images were captured using a Zeiss LSM 700 confocal microscope (Zeiss LSM 700, Carl Zeiss, Oberkochen, Germany).

### 2.6. Cellular Uptake Assay

The rNSPCs were seeded in a 24-well plate at 1.25 × 10^5^ cells/mL and co-cultured with 320 µg/mL of PEG-GQDs for 0 h, 2 h, 4 h, 16 h, and 24 h. The cells were then washed with PBS three times, detached using Accutase (Millipore, Burlington, MA, USA, SCR005), and then resuspended in cell staining buffer (Biolegend, San Diego, CA, USA, 420201). The uptake of PEG-GQDs into rNSPCs was analyzed by fluorescence-activated cell sorting (FACS) analysis (Beckman Cytoflex, Brea, CA, USA), based on the blue fluorescence signals of PEG-GQDs.

### 2.7. Immunocytochemistry

For identifying the stemness and differentiation potential of primary culture rNSPCs, immunocytochemical experiments were performed and the data are shown in Figure S3. In detail, after culture is placed in growth media for two days or differentiation for seven days, rNSPCs were fixed for 30 min in 4% PFA, permeabilized with 0.05% Triton X-100 in PBS solution for 10 min, and then blocked with 1% BSA in PBS at room temperature for 1 h. The cells were incubated with primary antibodies diluted with 1% BSA overnight at 4 °C. The primary antibodies used were anti-Nestin (Millipore, Burlington, MA, USA, MAB353, 1:200), anti-β-tubulin III (Tuj1) (Covance, San Diego, CA, PRB-435P, 1:250), Glial fibrillary acidic protein (GFAP) (Dako Cytomation, Carpinteria, CA, Z0334, 1:500), and Olig-2 (Millipore, Burlington, MA, USA, MAB9610, 1:200). After washing three times in PBS, the rNSPCs were incubated with secondary antibodies diluted with 1% BSA for 1 h at room temperature. FITC-conjugated secondary antibodies (Jackson Immuno Research, West grove, PA, USA, 715-095-150 and 711-095-152, 1:250) were used. At the last 15 min, cells were counterstained with Hoechst (Invitrogen, Eugene, OR, USA, H1399, 1:1000,) and washed with PBS three times. Imaging was performed using a fluorescence microscope (Olympus IX7151, Tokyo, Japan).

For quantitative analysis, Tuj1 and GFAP-positive cells were manually quantified in a visual field at 400× (*n* = 15). The total cell numbers were quantified by Hoechst staining.

### 2.8. Statistical Analysis

All results are presented as means ± standard deviations (SD). For all assays, one-way ANOVA and Tukey’s comparison tests were performed to determine possible significant differences (*p* < 0.05) between groups.

## 3. Results and Discussion

### 3.1. PEG-Graphene Quantum Dot (PEG-GQDs) Characterization

**Figure 1:**
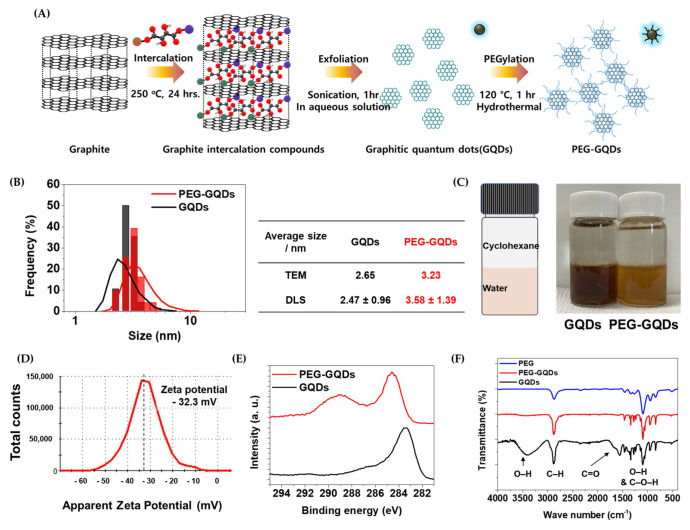
(**A**) Schematic illustration of the GQDs and PEG-GQDs fabrication. (**B**) Particle size distribution taken from transmission electron microscope (TEM) images (bar) and Dynamic light scattering (DLS, line) of the GQDs and PEG-GQDs. (**C**) Photograph of cyclohexane/water hydrophilicity/miscibility test. (**D**) Zeta potential of PEG-GQDs. (**E**) X-ray photoelectron spectroscopy (XPS) C 1s peaks. (**F**) Fourier-transform infrared spectroscopy (FTIR) of the PEG, GQDs, and PEG-GQDs.

As illustrated in Figure 1A, GQDs were fabricated by exfoliation using the GICs method, which is known as an efficient and mass-producible method to fabricate high-quality GQDs. By using this method, the resulting particles generally showed narrow size distribution. This is a significant point because size is the main parameter of controlling the photoluminescence property in quantum dots. The well-synthesized GQDs showed a narrow size distribution and exhibited characteristic photoluminescence under UV light. Therefore, it was confirmed that the GQDs can be used for tracing the pathway of the materials and there were no distinct differences in an optical property before and after PEGylation. The detailed PL properties are reported in previous papers [35,41].

The internalization of GQDs into the cells must precede to track the behavior of stem cells thoroughly. During the internalization process, the size of the materials is essential information for expecting the internalizing mechanism. The particle sizes were examined by using two different methods of TEM and DLS analysis (Figure 1B bar—TEM and line—DLS, respectively, and Figure S1 showed TEM analysis). The average size of GQD particles observed from the TEM was approximately 2.65 nm with a narrow size distribution similar to the value from the DLS method. After the PEGylation, the size of encapsulated particles was slightly increased to 3.23 nm from the TEM analysis. However, in the solution, the hydrophilic polymer layer remained hydrated. The hydrodynamic diameter of the PEG-GQDs was 3.58 nm, which is larger than the value from the TEM analysis. The PEG layer thickness in the aqueous solution was around 0.55 nm on the GQD particles. Consequently, it is expected that the PEG-GQDs particles are internalized into the cell through the passive penetration mechanism [42].

Moreover, the obtained GQDs and PEG-GQDs showed water-soluble properties with highly stable dispersion, as shown in Figure 1C. The zeta potential of the PEG-GQDs showed a strongly negative value of −32 ± 7 mV. (Figure 1D) The high negative value indicates that the colloidal solution is electrostatically stable. Furthermore, the PEG layer on the GQDs made it more stable in a steric way since the particles could effectively reduce the aggregation and biofouling effect [43].

The XPS spectra of carbon 1s were investigated to obtain more information about the surface functional groups. As shown in Figure 1E, the C1s spectrum confirmed the presence of the carbon species sp^2^ (~284 eV), sp^3^ (~284.8 eV), and C–O or C–OH (~286.6 eV); and functional groups amide (N–C=O) (~288.7 eV), carboxyl (COOH), or ester (~290 eV), respectively. The binding energy of GQDs presented a predominant peak near 284 eV. This is close to the sp2 binding energy with an asymmetric shape, which is preferably derived from graphite or graphene samples. After the PEG coating, the portion of sp3 was increased. Moreover, the peak of the PEG and amide group was revealed at around 286.6 and 289 eV. These XPS results confirm the presence of PEG on the GQDs after successful encapsulation.

This result was also crosschecked by the FT-IR spectra analysis and is shown in Figure 1F. The chemical bonding on the surface was screened from the second derivate of the spectra and is assigned as C–O stretching ~1100 cm^−1^; C=C, C=O stretching, ester, aliphatic COOH, and conjugated C=O ~1600 cm^−1^; C-H stretching ~3000 cm^−1^; and O–H stretching ~3500 cm^−1^, respectively. After surface modification, the distinctively changed locations were highlighted. The C–O stretching epoxide peak at 1024 cm^−1^ appeared prominently in PEG-GQDs. The ratio between C-H stretching band (~2880 cm^−1^) and the C–O stretching band (1130 cm^−1^) was also changed after the PEGylation. On the surface of PEG-GQDs, the ratio of the C–H/C–O was lowered and the carboxyl groups at 1660 cm^−1^ completely disappeared. These results are evidence of the successful modification of the PEG layer on the GQDs.

In summary, it was confirmed that the PEG modification successfully proceeded on the GQDs surface. It is expected that they could increase the utilization of nanoparticles as a bio labeling probe without an accumulation of unwanted locations caused by nonspecific binding on the cell and proteins and simultaneously reduce the biological interaction than the pristine GQDs.

### 3.2. Visualization Ability of PEG-GQDs

**Figure 2:**
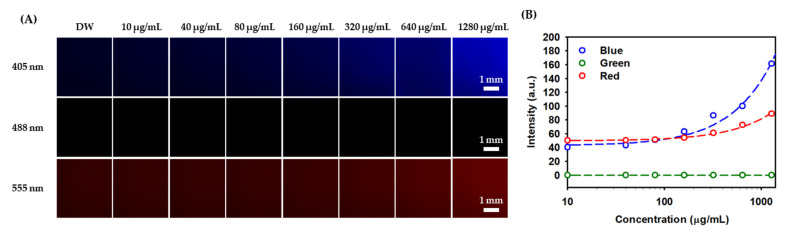
Optical fluorescence of PEG-GQDs prepared in DW at various concentrations and visualized at three different wavelengths (405 nm, 488 nm, and 555 nm). (**A**) Optical fluorescence images of the PEG-GQDs at different exposure times (405 nm: 1000 ms; 488 nm: 1500 ms; 555 nm: 700 ms). (**B**) The quantification figure of the fluorescence intensity for the PEG-GQDs at different exposure time.

The samples were prepared at various concentrations in the DW to investigate the visualization ability of the PEG-GQDs. The images of PEG-GQDs were captured with a fluorescence microscope with three different wavelengths. As shown in Figure 2, depending on the incident light source, the intensity and location of the signal were visualized by a pseudo-color RGB to assist comprehension. Due to the different yield ratios of blue, green, and red fluorescence signals, the different and same exposure time images were taken and shown in Figure 2 and Figure S2, respectively. Among them, the blue fluorescence signals of PEG-GQDs were the strongest. The fluorescence signals were shown a dose-dependent variation from 0 mg/mL to 1.28 mg/mL. These values could be used to predict the PEG-GQDs concentration in live cells. Furthermore, it would be useful as a standard for predicting the concentration of delivered drugs into cells or tracking implanted cells in clinical applications.

### 3.3. In Vitro Biocompatibility of PEG-GQDs

In neural regeneration, neural stem/progenitor cells (NSPCs) are one of the major cell types for stem cell based therapies because the NSPCs’ self-renewing and multipotent characteristics can generate the critical phenotype cells of the nervous system such as neurons, astrocytes, and oligodendrocytes [44,45,46,47]. In this study, embryonic NSPCs were used for investigating the biocompatibility and cytotoxicity of PEG-GQDs.

**Figure 3:**
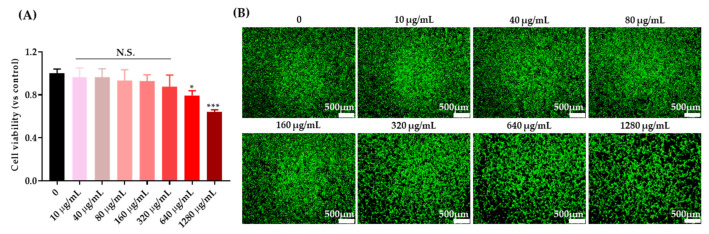
Cytotoxicity test of PEG-GQDs in rat neural stem/progenitor cells (rNPSCs). The amounts of 10,000 rNPSCs were seeded on the Matrigel pre-coated 96-well plate. After the cells grew for 24 h, the gradient concentrations of PEG-GQDs were added to the cells. The series of cell viability tests were then checked by a CCK assay (**A**) and live-dead staining (**B**) after 24 h of incubation. (**A**) In the Cell counting Kit-8 (CCK-8) assay, the rNSPCs were treated with CCK-8 reagent diluted with prewarmed media and incubated for 2 h at 37 °C. Then, the absorbance values were measured with a microplate reader at 450 nm. (**B**) For the live/dead assay, we used the live/dead viability cytotoxicity kit. The cells were incubated with Calcein AM and Ethidium homodimer-1 for 30 min. Live cells were distinguished by the green fluorescence of Calcein AM and dead cells were distinguished by the red fluorescence of Ethidium homodimer-1. Then, images were obtained with a fluorescence microscope at 40×. Scale bar: 500 µm. The error bars represent the standard deviation of the mean (*n* = 3). “*” indicates a significant difference between the control and experimental groups (*p* < 0.05). “***” indicates a significant difference between the control and experimental groups (*p* < 0.001).

After seeding rNSPC for 24 h, various concentrations of PEG-GQDs (0, 10, 40, 80, 160, 320, 640, and 1280 µg/mL) were added and then incubated with cells for an additional 24 h. As shown in Figure 3, the cell viability and live-dead staining pictures indicated that the PEG-GQDs did not show noticeable toxicity to rNSPCs up to 320 µg/mL. It is consistent with other researchers’ studies [22,23]. Yu Chong et al. [22] also reported the positive biocompatibility of GQD–PEG in vivo even under a multi-dosing situation. It indicated the possibility of applying PEG-GQDs for neural regeneration and even further clinical studies.

### 3.4. Cellular Uptake of PEG-GQDs

**Figure 4:**
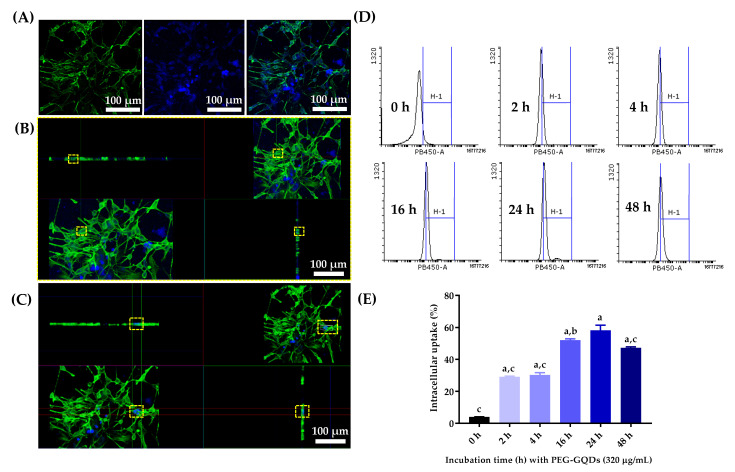
The absorption and intracellular location of PEG-GQDs in rat neural stem/progenitor cells (rNPSCs). (**A**) Confocal microscopy imaging of PEG-GQDs (320 µg/mL) in rNSPC cells. (**B**) Fewer PEG-GQDs were absorbed in the cell. (**C**) The aggregated PEG-GQDs were observed in the cell. The yellow square represents the region of interest. Green fluorescence was cell body stained with Phalloidin and blue fluorescence was from PEG-GQDs. (**D**) The rNSPCs were incubated with or without 320 µg/mL PEG-GQDs for 0 h, 2 h, 4 h, 16 h, 24 h, and 48 h at 37 °C, 5% CO_2_, and 100% humidity incubator. As the incubation time increased, more cells were detected with the PEG-GQDs (320 µg/mL) by the Fluorescence-activated cell sorting (FACS) method. The Y coordinate axes represent the count of cell number. (**E**) The percentage of cells detected with PB450-A fluorescence signals of PEG-GQDs (320 µg/mL) in rNSPC cells were summarized. The uptake of PEG-GQDs increased over the time and reached saturation after 24 h. Scale bar: 50 µm. The error bars represent the standard deviation of the mean (*n* = 3). “a” indicates a significant difference between the 0 h and experimental groups (*p* < 0.0001). “b” indicates a significant difference between the 24 h and experimental groups (*p* < 0.005). “c” indicates a significant difference between the 24 h and experimental groups (*p* < 0.0001).

Figure 4A–C demonstrated the confocal microscopy images of the internalized PEG-GQDs and rNSPCs after 24 h incubation. The rNSPCs internalized the PEG-GQDs and showed fluorescence signals. The blue fluorescence signal comes from the PEG-GQDs, and the green fluorescence signal comes from the stained F-actin of the cells. The blue signal from PEG-GQDs was located near the green signals from the cell body and the PEG-GQDs were frequently observed inside the cells. The bio labeling of PEG-GQDs in live rNSPCs was shown in Figure S4. Figure 4C showed the strong blue signals co-localized with the cell body. It seems that highly concentrated PEG-GQDs are rarely found inside the cells. Nearly 95% of cells did not show aggregated PEG-GQDs inside the cell body. It was revealed that the PEG-GQDs were well internalized inside the cell body and had good biocompatibility without severe aggregation.

Fluorescence-positive cells were sorted by performing FACS analysis to quantify the rate of intracellular uptake ratio of PEG-GQD in rNSPC with respect to incubation time. Percentages of blue fluorescence-positive cells were then calculated based on the FACS analysis. As shown in Figure 4 D, E, the uptake amount of PEG-GQDs increased over time and the value reached saturation after 24 h. In the work conducted by Weihu Shang et al. [21], it was also reported that GQD uptake in hNSCs was relatively fast and occurred in a time-dependent manner. Around 40% of the cells were not detected by fluorescence signals, suggesting that PEG-GQDs did not affect rNSPCs’ self-renewal and proliferation ability.

### 3.5. PEG-GQDs Do Not Affect the Differentiation Potential of rNSPCs

**Figure 5:**
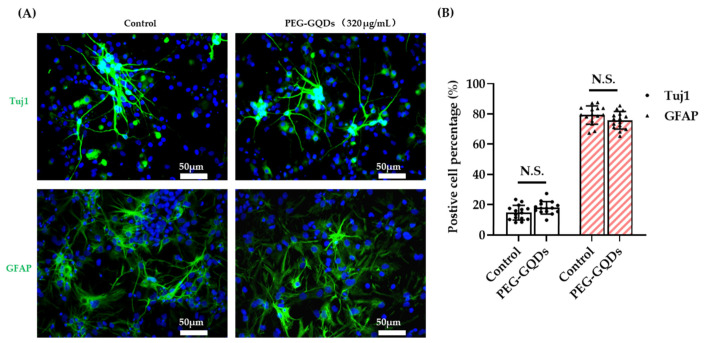
PEG-GQDs did not affect the differentiation potential of rat neural stem/progenitor cells (rNPSCs). (**A**) The rNSPCs were incubated for 24 h without or with 320 µg/mL PEG-GQDs. Subsequently, the growth media was replaced with Neurobasal plus media to induce differentiation. After 7 days of differentiation, immunocytochemistry assays were performed to estimate the differentiation potential of rNSPCs. Representative microphotographs demonstrated β tubulin III (Tuj1)- and Glial fibrillary acidic protein (GFAP)-positive cells. The nuclei were counterstained with Hoechst (blue). Scale bar: 50 µm. (**B**) The percentage of Tuj1- and GFAP-positive cells were normalized with Hoechst- positive total cells numbers. There was no significant difference compared with the non-treated control group. “N.S.” indicates no difference between the control and experimental groups (*n* = 15, *p* > 0.05).

The differentiation potential of rNSPCs with internalized nanomaterials is an essential characteristic for stem cell based therapy that should not be changed by the containing material for further bio applications. To investigate whether PEG-GQDs impair the differentiation potential of rNSPCs, we induced a differentiation process of rNSPCs. After 7 days of differentiation, neuron-specific class III beta-tubulin (Tuj1) and glial fibrillary acidic protein (GFAP) were examined using immunocytochemistry. As shown in Figure 5, no significant difference was observed between the control and 320 µg/mL PEG-GQDs treated groups. The rNSPCs with the PEG-GQDs were also able to differentiate into neurons and glial cells (Figure 5A). Moreover, Tuj1 positive stained neurons showed good morphology of elongated cell shapes with long neurite outgrowth and formed an excellent interconnected neuronal network [48]. Moreover, there were no notable differences in the percentages of neurons or glial cells between the two groups (Figure 5B). The results demonstrated that 320 µg/mL PEG-GQDs treatment did not affect the differentiation process of rNSPCs towards both neurons and glial cells. The results in this study were consistent with the study of Weihu Shang et al. They reported that 25 µg/mL GQD treatment did not affect the differentiation percentages of both neurons and glial cells of human neural stem cells [21]. It is obvious that our PEG-GQDs did not influence the neural stem cell differentiation even at the concentration of the materials 10 times higher than the previous reports.

The successful PEGylation did not interfere with the photoluminescence properties of GQDs while passivating the surface from other reactive substances. The assays related to the biological effects on rNSPCs showed that the PEG-GQDs exhibited excellent biocompatibilities, bioimaging property without weakening the rNSPCs’ activity, and differentiation ability under concentrations up to 320 µg/mL. Moreover, the PEGylation of GQDs may extend the in vivo circulation time and improve the penetration efficiency to nerve tissue, leading to enhanced bio-imaging, delivering, and therapeutic effects to the nerve tissue injury sites.

## 4. Conclusions

It was confirmed that the interaction between PEG-GQDs and rNSPCs may provide some useful information for future explorations of a biocompatible and visible on-target probe for the different therapies for neurological disorders. The uniform-sized, water-soluble, and light-emitting GQDs are successfully combined with the PEG that is used to enhance the colloidal stability with a hydrophilic surface modification. Our approach takes advantage of the photoluminescence property of GQDs without distinct losses and takes advantage of the non-reactive hydrophilic surface of the PEGs. The synthesized material showed no specific cytotoxicity up to a concentration of 320 µg/mL and exhibited an ability to be imaged when the material was internalized inside the cells. The embryonic rNSPCs that internalized the substances were investigated on whether they sustain their differentiation ability. The result showed that the differentiation potentials of rNSPCs were not adversely affected by PEG-GQDs.

In conclusion, PEG-GQDs showed proper bio labeling ability and biocompatibility without attenuation of rNSPCs differentiation, suggesting that PEG-GQDs are useful material tools at the visible nano-delivery system for the regeneration therapies against neurological disorders.

## Data Availability

The data presented in this study are available in insert article and supplementary material here.

## Supplementary Materials

The following are available online at https://www.mdpi.com/article/10.3390/nano11061446/s1, Figure S1: (A) X-ray Diffraction (XRD) pattern and Transmission electron microscope (TEM) images; Figure S2: Optical fluorescence of PEG-GQDs prepared in DW at various concentrations, visualized at three different wavelengths (405 nm, 488 nm and 555 nm) with same exposure time; Figure S3: Identification of rat neural stem/progenitor cells (rNSPCs); Figure S4: (A) The video of 3D condition of absorption and intracellular location of PEG-GQDs in rat neural stem/progenitor cells (rNPSCs), (B) Optical fluorescence images of the PEG-GQDs (320 µg/mL) in living rNPSCs.
