# Supplementary material for: Biological Potential of Polyethylene Glycol (PEG)-Functionalized Graphene Quantum Dots in In Vitro Neural Stem/Progenitor Cells
Source: Nanomaterials (Basel). 2021 May 29;11(6):1446. doi: 10.3390/nano11061446 (PMC8226482; doi:10.3390/nano11061446)
Supplement: Supplementary file 1 [file nanomaterials-11-01446-s001.zip › nanomaterials-1220102-supplementary.pdf]

nanomaterials

Supplementary information

Biological potential of polyethylene glycol (PEG)-functionalized Graphene quantum dots in in-vitro Neural stem/progenitor cells

Yunseong Ji [1,2,†], Yu-Meng Li [1,2,†], Jin Gwan Seo [3], Taesu Jang [4], Jonathan Campbell Knowles [5,6,7], Sung Ho Song [3,*] and Jung-Hwan Lee [1,2,8,*]

[1] Institute of Tissue Regeneration Engineering (ITREN), Dankook University, 119 Dandae-ro, Cheonan, Chungcheongnam-do 31116, Korea; jys1432@naver.com (Y.J.); wendyleeym0925@gmail.com (Y.-M.L.)
[2] Department of Nanobiomedical Science & BK21 PLUS NBM Global Research Center for Regenerative Medicine, Dankook University, 119 Dandae-ro, Cheonan, Chungcheongnam-do 31116, Korea
[3] Division of Advanced Materials Engineering, Kongju National University, Cheonan, Chungnam 32588, Korea; wlsrhks0324@smail.kongju.ac.kr
[4] Department of Pre-medi, College of Medicine, Dankook University, Cheonan 31116, Korea; jangts@dankook.ac.kr
[5] UCL Eastman-Korea Dental Medicine Innovation Centre, Dankook University, 119 Dandae-ro, Cheonan 31116, Korea; j.knowles@ucl.ac.uk
[6] Division of Biomaterials and Tissue Engineering, Eastman Dental Institute, University College London, London WC1E 6HH, UK
[7] The Discoveries Centre for Regenerative and Precision Medicine, Eastman Dental Institute, University College London, London WC1E 6HH, UK
[8] Department of Regenerative Dental Medicine, College of Dentistry, Dankook University, Cheonan, Chungcheongnam-do 31116, Korea
* Correspondence: shsong805@kongju.ac.kr (S.-H.S.); ducious@gmail.com (J.-H.L.); Tel.: +82-41-521-9379 (S.H.S.); Fax: +82-41-521-5776 (S.H.S.); Tel.: +82-41-550-3081 (J.-H.L.); Fax: +82-41-559-7839 (J.-H.L.)
† These authors contributed equally to this work as first authors.

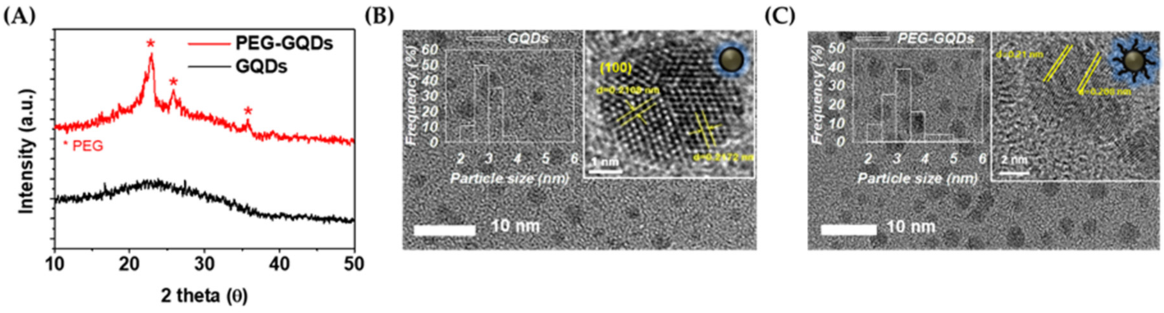

Figure S1. (A) X-ray Diffraction (XRD) pattern and Transmission electron microscope (TEM) images (inset; particle size distribution histogram and high magnification images corresponding to specific lattice space of graphitic structure). (B) GQDs and (C) PEG-GQDs. [RSC Adv., 2020, 10, 27418–27423] Figure S1 images were reproduced with permission copyright 2020 Royal Society of Chemistry.

Highly luminescent polyethylene glycol-passivated graphene quantum dots for light emitting diodes

H. J. Kim, C. K. Lee, J. G. Seo, S. J. Hong, G. Song, J. Lee, C. Ahn, D. J. Lee and S. H. Song, *RSC Adv.*, 2020, **10**, 27418

DOI: 10.1039/D0RA02257H

This article is licensed under a Creative Commons Attribution 3.0 Unported Licence. Material from this article can be used in other publications provided that the correct acknowledgement is given with the reproduced material.
[Original citation] - Published by The Royal Society of Chemistry.

Chemical oxidation carves the sharp corners and makes materials round like a pebble at the rivers. Therefore, the shape of the GQDs is mostly circular which was observed from the TEM and the diameter was around 2.65 nm. They were torn down from the graphite and not much changed from their original graphitic structure. The height of the GQDs was 1-1.5 nm confirmed by the AFM. This means the particles are squashed-spherical shaped and composed of 3-5 layered graphitic quantum dots. (The lattice space of the pristine graphite is 0.335 nm and d-value of GQDs from XRD was around 0.358 nm.)

Since the GQDs were made by the top-bottom method, they usually experience size reduction and chemical structure distortion. Because of the small size, the characteristic peak of the graphitic structure was not clearly observed in XRD (Figure S1A), but the characteristic lattice space of (001) of graphite is observed in TEM (Figure S1B and C inset). This revealed that the structure of GQDs originated from the graphite structure. From these results, the peak shown in XRD was confirmed that the broad peak represents the (002) peak of GQD.

The average size of the resulting particles was 2.65 nm with a narrow size distribution. As well, after the PEGylation, there was no significant difference in the crystal structure from the pristine GQDs. And the appearance of the PEG peak was observed in XRD and the size of encapsulated particles was observed that slightly increased to 3.23 nm in TEM.

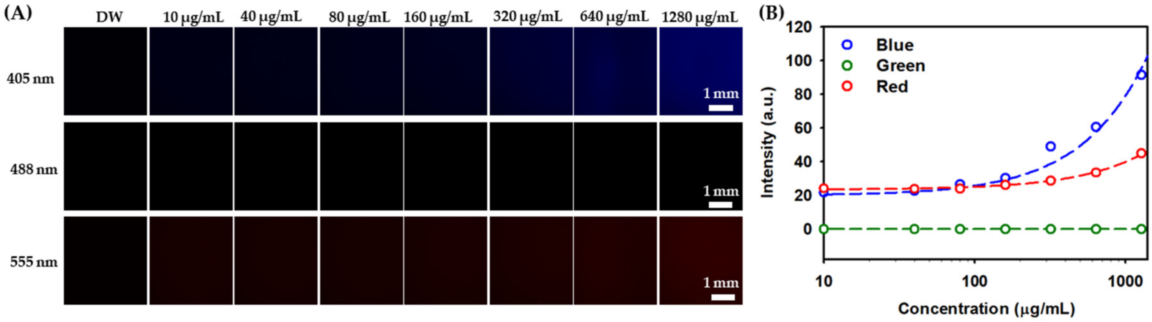

Figure S2. Optical fluorescence of PEG-GQDs prepared in DW at various concentrations, visualized at three different wavelengths (405 nm, 488 nm and 555 nm) with same exposure time. (A) Optical fluorescence images of the PEG-GQDs at same exposure time: 200ms; (B). The fluorescence intensity quantification figure of the PEG-GQDs at same exposure time.

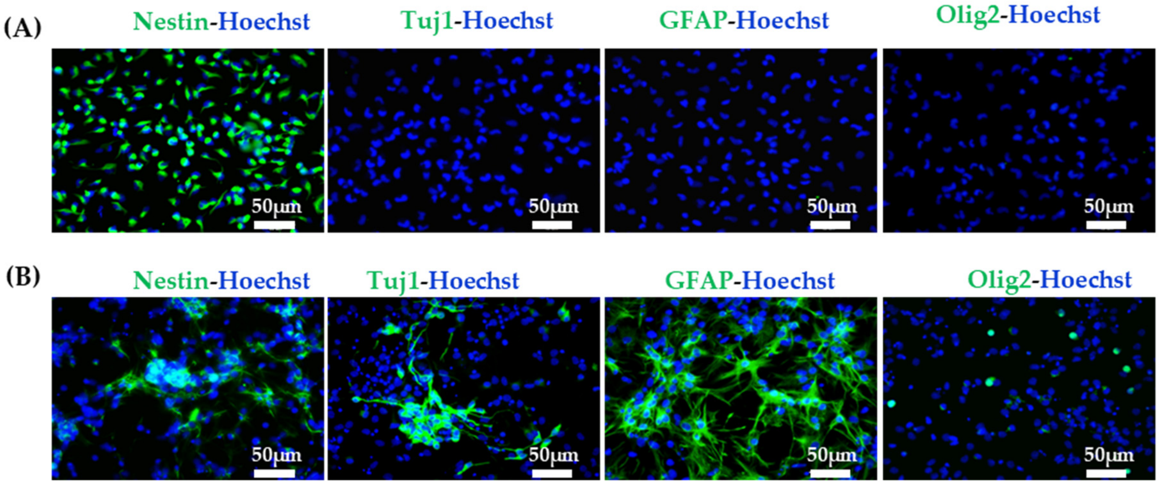

Figure S3. Identification of rat neural stem/progenitor cells (rNSPCs). (A) neural stem cell makers and differentiation makers staining for identification the stemness of rat stem/progenitor cells; (B) Differentiation abilities of rNSPCs via fluorescence staining of neuronal maker β tubulin III (Tuj1), astrocyte maker GFAP and oligodendrocyte marker Olig2 after 7 day's culture in differentiation media. Scale bar = 50 μm.

(A) Figure S4 (A) video.mp4

(B)

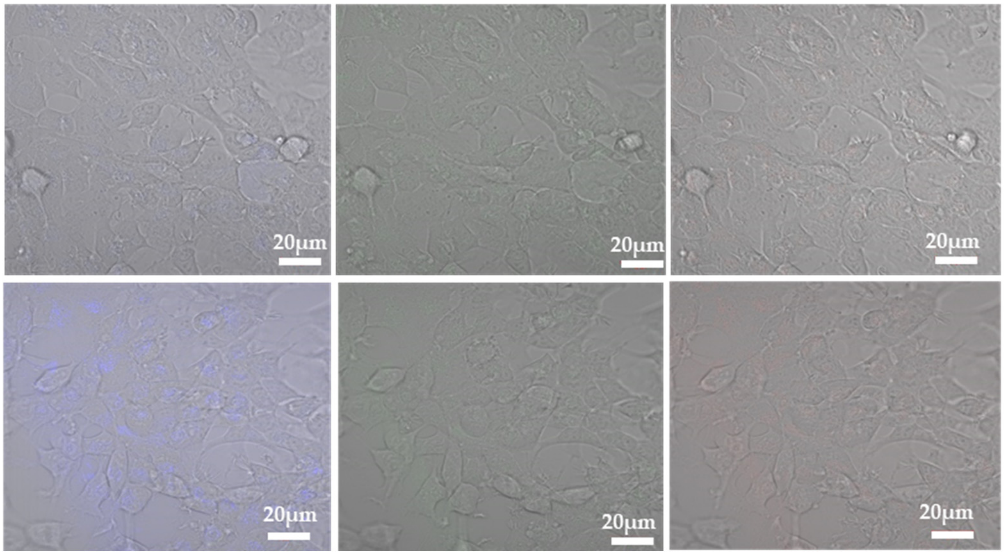

Figure S4. (A) The video of 3D condition of absorption and intracellular location of PEG-GQDs in rat neural stem/progenitor cells (rNPSCs); Green fluorescence comes from the Phalloidin stained F-actin, blue fluorescence comes from the PEG-GQDs; (B) Optical fluorescence images of the PEG-GQDs (320 μg/mL) in living rNPSCs after 24 hours incubation, visualized at three different wavelengths (405 nm, 488 nm and 555 nm). Scale bar = 20 μm.
